# Efficacy of Ginger in the Treatment of Primary Dysmenorrhea: A Systematic Review and Meta-analysis

**DOI:** 10.7759/cureus.13743

**Published:** 2021-03-06

**Authors:** Rizu Negi, Suresh K Sharma, Rakhi Gaur, Anupama Bahadur, Prasuna Jelly

**Affiliations:** 1 Obstetrics and Gynecology, All India Institute of Medical Sciences, Dehradun, IND; 2 College of Nursing, All India Institute of Medical Sciences, Jodhpur, IND; 3 Obstetrics and Gynecology, Akal College of Nursing, Eternal University, Himachal Pradesh, IND; 4 Obstetrics and Gynecology, All India Institute of Medical Sciences Rishikesh, Rishikesh, IND

**Keywords:** ginger, primary dysmenorrhea, zingiber, systematic review, menstrual pain

## Abstract

It has been evidenced that very few systematic reviews have examined the effectiveness of ginger for pain duration and its severity among women with primary dysmenorrhea. This meta-analysis was therefore performed to methodically incorporate and significantly evaluate randomized controlled ginger studies for the treatment of primary dysmenorrhea. The literature was searched using PubMed, Embase, Ovid, ClinicalKey, Medline, and electronic database. We have analyzed clinical trials by comparing ginger with placebo and non-steroidal anti-inflammatory drugs in women with primary dysmenorrhea. The primary outcomes assessed in our meta-analysis were pain severity and pain duration. Secondary outcomes were change in bleeding, side effects of the drug, and rate of satisfaction. We have screened a total of 638 studies, out of which narrative synthesis was formulated for eight studies. We have performed a meta-analysis of five trials examining ginger with placebo and other two randomized controlled trials comparing ginger with a non-steroidal anti-inflammatory drug (NSAID); it seems to be more helpful for relieving menstrual pain than a placebo (mean difference [MD] = 2.67, 95% CI = 3.51-1.84, P = 0.0001, I2 = 86%), although it was found that ginger and NSAIDs were equally effective in pain severity (risk ratios [RR] = 1.15, 95% CI = 0.53-2.52, P = 0.72, I2 =77%). We have not observed any significant difference between ginger and placebo on pain duration among primary dysmenorrheic women (MD = -2.22, 95% CI = -7.62-3.18, P = 0.42, I2^ ^= 56%). Accessible information proposes that oral ginger can be a compelling treatment for primary dysmenorrhea. This meta-analysis strongly supports the requirement for high methodological quality consistency for upcoming trials.

## Introduction

Primary dysmenorrhea is a common condition that occurs in the absence of any pelvic disease. It is one of the most familiar gynecology problem, which decreases the performance of women and causes 34%-50% of absentees from education and career; dysmenorrhea has many social and economic ramifications, and this impacts the psychological health of women along with the quality of life [1-3]. Dysmenorrhea results from the withdrawal of progesterone near the peak of a menstrual cycle; this withdrawal has been shown to extend the synthesis of prostaglandins F2 (PGF2) and E2 (PGE2). Awed et al.'s study suggests that prostaglandins are released during menstruation because of endometrial cell destruction. PGE2 stimulates uterine contractions and increases vasopressin release, which ends up in ischemia and pain [4].

Berkley’s study [5] mentioned that dysmenorrhea has been distinguished as primary and secondary. In the absence of pelvic conditions, primary dysmenorrhea occurs when the pain starts at the beginning of menstrual bleeding and continues for 12-48 hours. Akhlaghi et al.’s study [6] found that the prevalence of dysmenorrhea is mentioned in many studies, and it varies between 50% and 90%. Non-steroidal anti-inflammatory medications are the standard medication for dysmenorrhea and have many side effects, such as headache, giddiness, dysuria, fatigue, anorexia, vomiting, skin inflammation, and gastric ulcer. Many studies have shown that herbal medication pain relief is much more practical than chemical drugs, and Rosenwaks and Seegar-Jones’s study [7] mentions that ginger is known as a complementary remedy to serve the purpose. Dugasani et al.'s study [8] revealed that herbs and spices are the various treatments used by women, which are widely accepted safe and known to be effective.

Traditionally, a range of folk medicine has been used to treat every day minor ailments such as menstrual cramps, headache, vomiting, indigestion, and nausea. Ginger is known to have outweighing benefits among many conventional remedies. It is useful in minimizing menstrual cramps, and it relaxes the muscular spasms as well. It is considered as an anti-inflammatory agent in folk remedies. It also contains non-volatile components like gingerols, shogaols, zingerone, and paradol. Furthermore, it has pleiotropic pharmacological activities, like antioxidants, under the prolonged exposure of the desensitized TRPV1 agonists, capsaicin and shogaol, which ends in pain relief [8-10].

Few systematic reviews have investigated the effectiveness of ginger in general pain relief (acute and chronic pain, including only a few early ginger-specific trials in primary dysmenorrhea) [11,12]. There were some studies that have compared ginger with placebo and showed beneficial effects in primary dysmenorrhea [1]. In 2016, Chen et al.’s systematic review [12] of ginger efficacy was reported for primary dysmenorrhea, but with limited trials, and findings were also restricted by some factors such as the inclusion of non-randomized controlled trials (RCT) studies. Therefore, an additional thorough and accurate systematic review and meta-analysis were needed. The objective of this review was to systemically analyze all randomized clinical studies of the effect of ginger on the treatment of primary dysmenorrhea and to explain its effectiveness in alleviating the symptoms of primary dysmenorrhea with the well-framed research question: Is oral ginger (intervention) successful in minimizing menstrual pain (main outcome) in women with primary dysmenorrhea compared to placebo or non-steroidal anti-inflammatory drug (NSAID) (comparison) (population)?

## Materials and methods

For this systematic study and meta-analysis, we have followed Preferred Reporting Items for Systematic Reviews and Meta-Analyses (PRISMA) guidelines. To answer the review question with justification, the PICO (patient/population, intervention, comparison and outcomes) framework was used. The primary outcomes for this review were pain severity and pain duration. Secondary outcomes were changes in bleeding, side effects of the drug, and rate of satisfaction.

Data sources and selection criteria

PubMed, Embase, Ovid, ClinicalKey, Medline, and electronic database were searched for data. The Medical Subject Headings (MeSH) were “Ginger,” (Primary) “Zingiber officinale,” “primary dysmenorrhea,” “menstruation pain,” “randomized,” “placebo,” “controlled trial,” (dysmenorrhea∗ OR menstruation pain∗ OR “period∗ pain” OR “painful period∗”), “Dysmenorrhea* OR Ginger OR Pharmacological*”, and “pain modalities.” Also the list of references from the selected studies was examined for the additional trials and evidence.

Study selection

Studies were searched independently and screened potentially for eligibility by two reviewers who read the title, abstract, and related references to select literature that requires a detailed examination. Whenever there is any difference in the opinion of two reviewers, then the third reviewer was approached to take the last decision. We also contacted the authors whose studies needed further clarity. In this review, RCTs comparing the effectiveness of oral ginger with placebo or non-steroidal anti-inflammatory medication (NSAID) in women with primary dysmenorrhea evaluated by a patient-reported outcome measure were included. RCTs from the year 2008 to 2020 published only in English language were considered for inclusion. Studies were excluded if they were non-RCTs, case control, cohort, letters, reviews, trials of ginger combined with other substances, and non-human or in vitro studies. The two reviewers who carried out the search examined the eligibility of the studies on the basis of the Joanna Briggs Institute (JBI) Critical Appraisal checklist for RCT, and the data is mentioned in Table 1.

**Table 1:** Study characteristics BD, Two times a day; TID, three times a day; QID, four times a day; JBI, Joanna Briggs Institute; VAS, visual analog scale.

Study	Participant Characteristics		Sample Size	Intervention (Ginger Powder)		Comparison		Outcome (Incidence)			Quality (JBI Score)
	Age (Years)	Pain Severity		Preparation	Dosage	Preparation	Dosage	Pain Severity		P value	
								Dichotomous	Continuous		
Rahnama et al., 2012 [1] (Single-Center Study)	>18	Moderate to severe	Ginger: 59 Placebo: 46	Ginger capsule (unknown origin and constituents)	500 mg, TID × 5 days	Placebo	500 mg, TID		Ginger: 5.12 ± 2.69; Placebo: 6.58 ± 2.02 (Pain duration)^*^; Ginger: 14.7 ± 18.36; Placebo: 21.36 ± 22.59	0.015 0.017	Moderate (10/13)
Shirvani et al., 2014 [14] (Single-Center Study)	>18	Pain intensity over 40 mm based on 100 mm VAS	Ginger: 61 Control: 61	Ginger capsule (Zintoma; Iranian brand)	250 mg, QID until the pain subsides	Mefenamic acid	250 mg, TID	Ginger: 27/60 Control: 33/60		>0.05	Low (5/13)
Ozgoli et al., 2008 [15] (Multicenter Study)	>18	Moderate to severe	Ginger: 50 Control: 50	Ginger capsule (Zintoma; Iranian brand)	250 mg, QID × 3 days	Ibuprofen	400 mg, QID	Ginger: 18/50 Control: 10/50	NA	>0.05	Moderate (8/13)
Kashefi et al., 2012 [16] (Multicenter Study)	15-18	Moderate to severe	Ginger: 47 Placebo: 45	Ginger capsule (unknown origin and constituents)	250 mg, TID × 4 days	Placebo	250 mg, TID		Ginger: 3.08 ± 1.52; Placebo: 6.95 ± 1.67	<0.001	High (11/13)
Abadi et al., 2020 [17] (Single-Center Study)	20-30	Not mentioned	Ginger: 70 Control: 70 Placebo: 70	Ginger capsule (unknown origin and constituents)	250 mg, QID × 3 days	Placebo	250 mg, QID		(Pain duration)^*^; Ginger: 1.61 ± 0.64; Placebo: 2.12 ± 0.81	0.052	Moderate (8/13)
Pakniat et al., 2019 [18] (Single-Center Study)	18-25	Moderate to severe	Ginger: 50 Control: 100 Placebo: 50	Ginger capsule (Zingiber officinale) (Ponstan; Razak Co., Tehran, Iran)	250 mg, BD × 3 days	Placebo	250 mg, BD		Ginger: 3.20 ± 1.28; Placebo: 6 ± 0.7	<0.001	Moderate (9/13)
Janebi et al., 2013 [19] (Single-Center Study)	>18	Moderate to severe	Ginger: 35 Placebo: 34	Ginger capsule (unknown origin and constituents)	500 mg, TID × 3 days	Placebo	Not mentioned		Ginger: 4.81± 1.7; Placebo: 7.11 ± 1.2	0.001	Moderate (9/13)
Rad et al. 2018 [20] (Single-Center Study)	18-26	Pain severity (grade 2 and grade 3)	Ginger: 78 Control: 90	Ginger capsule (unknown origin and constituents)	200 mg, QID × 2 days	Novafen	200 mg QID		Ginger: 3.10 ± 2.69; Control: 2.97 ± 2.69	>0.05	Moderate (10/13)

Outcome measures

Two authors collected a predefined outcome from the studies, which includes study characteristics and patients’ profiles. The primary study outcomes were pain severity and pain duration of primary dysmenorrhea. The secondary outcomes were changes in bleeding, side effects of the drug, and rate of satisfaction.

Data extraction

Data extraction was carried out by excluding duplicate studies. It has been done independently by three reviewers. The differences were resolute by the formal discussions and consensus by the senior reviewer. Data extraction forms were intended to tabulate the characteristics of the included studies. We have extracted the data of three reviewers and discussed the dataset with the fourth reviewer, who helped us resolve the discrepancies. Data on the subject of the first author, publication year, country, study type, population, interventions (ginger), outcomes (primary and secondary), and results were pulled. To get additional information, corresponding authors of the included studies were contacted through emails. We tried to contact four authors and only Shirvani et al. and Pakniat et al. reverted (mail) back.

Assessment of risk bias and quality assessment

Bias risks in the studies were evaluated by four authors autonomously. Risk bias evaluation was done by following the standard guidelines of Cochrane risk bias guidelines by Higgins et al. [13]. In case of any discrepancy between authors, the consultation from the fifth reviewer was considered to get the final decision. Risk of bias includes random sequence generation, allocation concealment, blinding, incomplete outcome data, selective reporting bias, and other bias. Four reviewers reviewed all included studies, and they used the Cochrane Collaboration approach for the assessment of risk bias. Two primary reviewers assessed for randomization bias, allocation concealment, blinding of participants and assessor, incomplete outcome data, and other bias. All studies were reported as low risk, high risk, and unclear risk for its bias toward each component. If a study reported a low risk for all domains of risk bias, it was considered to be of good quality and vice versa. If any dissimilar opinion arises between primary reviewers for risk bias, third and fourth reviewers did a thorough assessment of the study, and conclusions were made with mutual consensus.

**Figure 1:**
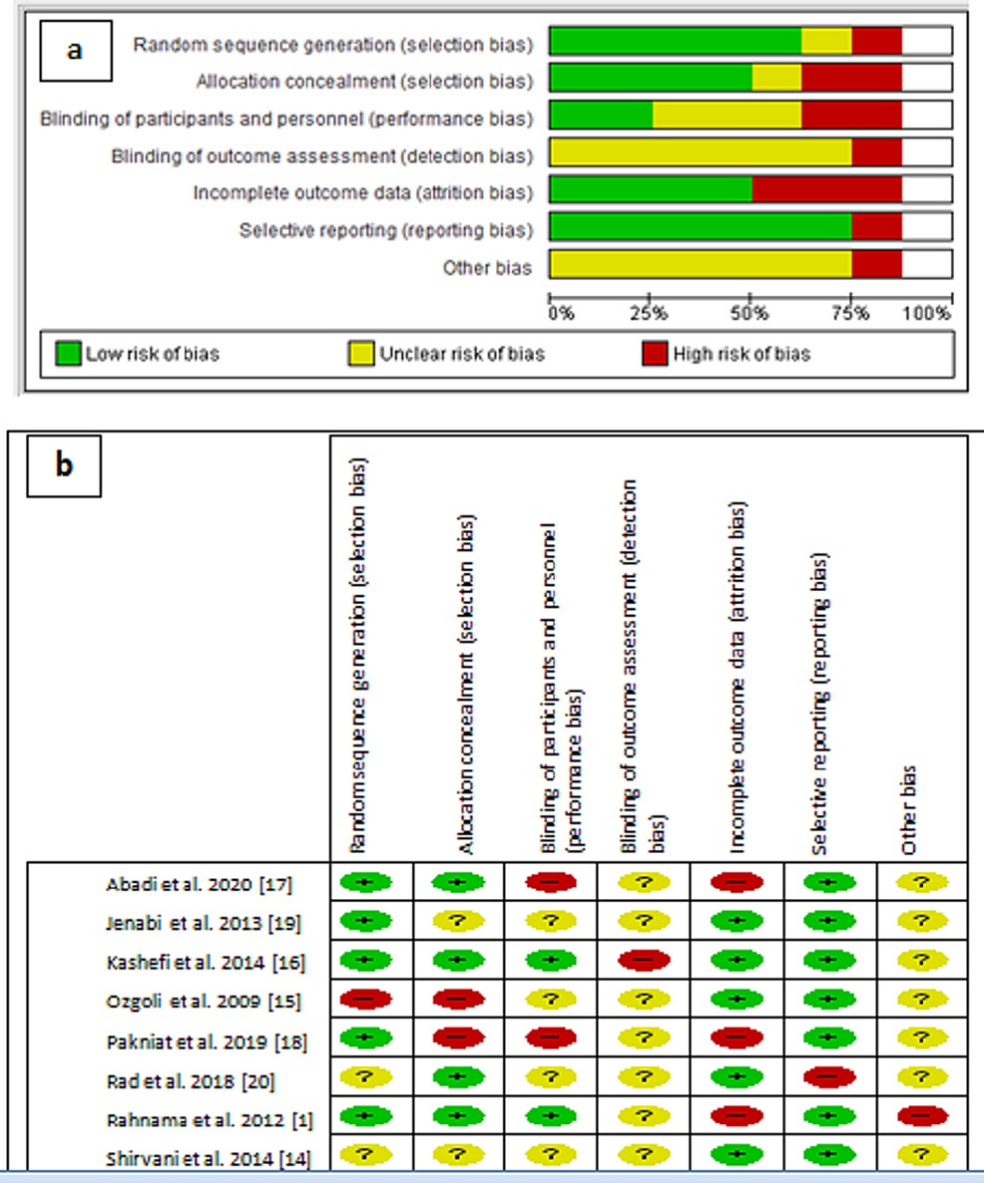
(a) Risks of bias graph. (b) Risks of bias summary.

The majority of the studies expressed a low to unclear level of risk bias (Figures 1a, 1b). For random sequence generation, five studies were decided as having a low-risk bias; Shirvani et al. [14] and Rad et al.'s studies were judged as unknown risk. Ozgoli et al.’s study [15] found a high-risk bias in the random sequence generation. In allocation concealment, Rahnama et al., Kashefi et al., Abadi et al., and Rad et al.’s studies were judged as low-risk bias [1,16,17]. Ozgoli et al. and Pakniat et al.’s studies [15,18] were criticized as having high-risk bias as participants were allocated alternatively into the groups. Two trials were judged as high-risk bias in blinding of participants and personnel [17,18]. Kashefi et al.’s study shows high-risk bias in the blinding of outcome assessment, and other remaining studies judged unclear in the bias detection. Attrition bias was found high in the Rahnama et al.’s study with other two studies [17,18].

In the selective reporting bias, only the Rad et al.’s study has high-risk bias as there was a mention of assessment of pain severity through pain visual analog scale (PVAS), but as a result, they changed the term from severity to intensity and have not mentioned it in the results table. All other studies fall in a low-risk bias in the selecting report. “Other risk bias” was considered high in Rahnama et al.'s study [1]; the result determined for Protocol 2 was biased by the effects of Protocol 1. For the remaining studies, “other risk of bias” were found unclear as a result of the restricted report. All the authors were consulted/informed in case of any missing information from the study findings, and after receiving the response from the corresponding authors of included studies, further decisions were made with the mutual consensus of all authors of this analysis. 

Data analysis

Narratively, data derived from the included studies were synthesized. Tabulation has been used to bring together trial features (i.e., author, participants, sample size, intervention, comparison, and outcome measures), designs across the studies analyzed in requisites of study characteristics, and its results. The aspect that may have altered the findings was further analyzed. Statistical research was carried out according to the statistical guidance protocol in the latest edition of the Cochrane Handbook for Systematic Review of RCT. RevMan Manager 5 (Nordic Cochrane Centre, Cochrane Collaboration, Copenhagen) was used for the production of review and data analysis.

In the present review, dichotomous outcomes (adding the impact of ginger and NSAIDs on pain severity) were represented as risk ratios (RR) with 95% confidence intervals (CI). Continuous results, such as symptom scores (e.g., as calculated by VAS), were expressed as mean difference (MD) with 95% CI. Heterogeneity was tested both by visual examination of forest plots (where non-overlapping CI suggested the probability of heterogeneity) and by the use of the Chi-squared heterogeneity test (differences at P < 0.05 are considered statistically significant). Heterogeneity was also represented as I2 figures, with a value of 0% suggesting no heterogeneity. Subgroup analysis to remove heterogeneity and funnel plots was not possible due to the small number of studies (<10). Furthermore, due to heterogeneity, we avoided a fixed model and performed statistical analysis using a random effect model for both continuous and dichotomous results.

## Results

Selection and characteristics of the studies

**Figure 2:**
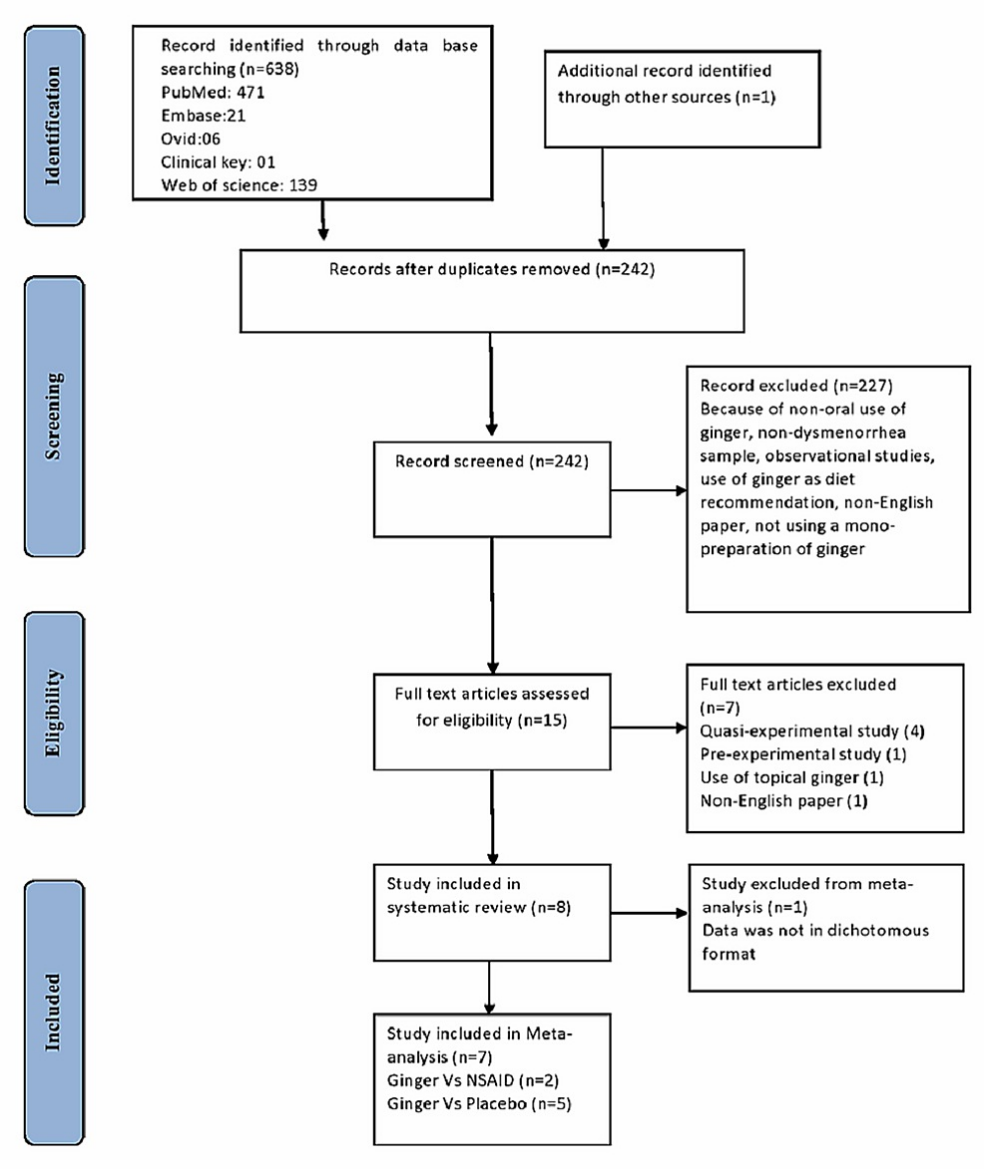
PRISMA flow diagram PRISMA, Preferred Reporting Items for Systematic Reviews and Meta-analyses.

A PRISMA flow diagram depicting the studies that are reported, screened, rejected, and included is shown in Figure 2. A total of 638 studies were listed in the initial electronic searches, and 396 duplicate studies were excluded. A total of 15 studies were found to be qualified under the title in which eight of the trials that met the requirements were comprised in the meta-analysis.

The comprised studies were conducted between 2008 and 2020. The main characteristics of the included studies are shown in Table 1. Seven of the eight included studies were of parallel design [1,14-19], and one study was having a cross-over design by Rad et al. [20]. All eight studies were conducted in Iran. Trial participants were either high-school or college students. In the included seven studies the outcome variable was pain severity, and in two studies the outcome variable was the duration of pain [1,18]. The sample size of the studies ranged from N = 70 to N = 201, and the ginger group lies between n = 35 and n = 78.

Each included trial tested ginger in a variety of dehydrated powder. The number of working components of ginger was not measured or recorded in any of the trials. Only Abadi et al.’s study mentioned the production of ginger and placebo capsules in Herbi Daru Pharmaceutical Company, Tabriz, Iran. The dose of ginger ranges from 700 mg to 1,000 mg per day. The common duration of treatment with ginger was the first to the third day of menstruation. A continuous numerical scale (visual analog scale) of 10 cm lines was used to evaluate pain severity in five trials. Only one of the studies measures pain severity by the multidimensional verbal scoring system (VMS) [15]. In Shirvani et al. [14] and Abadi et al.’s studies [17], “days in pain” data was collected for pain duration, whereas in Rahnama et al. [1], “hours in pain” data was collected.

Effects of intervention (outcome)

i. Pain severity

**Figure 3:**
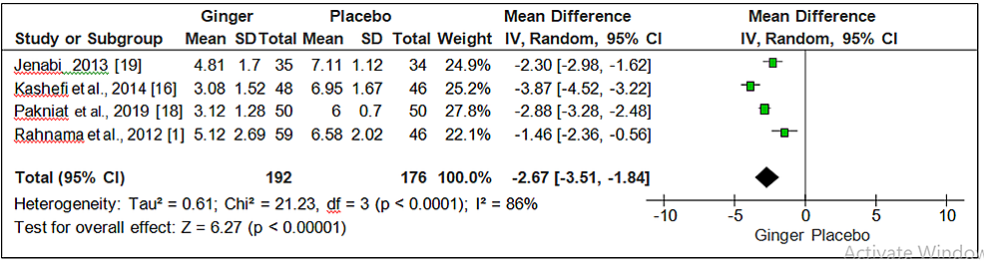
Forest plot for ginger versus placebo (pain severity)

Ginger versus placebo and ginger versus NSAID: Four studies observed the effect of ginger and placebo on pain severity on 245 participants associated with primary dysmenorrhea [1,16,18,19]. On meta-analysis, a significant reduction was found in the pain severity of participants in the ginger group compared to the placebo group (MD = 2.67, 95% CI = 3.51-1.84, P = 0.0001, I2 = 86%) (Figure 3).

**Figure 4:**
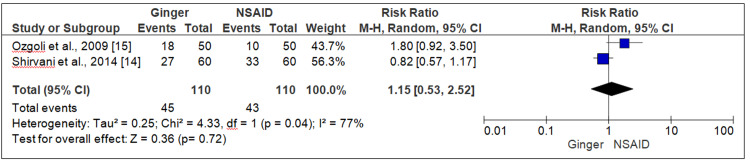
Forest plot ginger versus NSAID (pain severity) NSAID, Non-steroidal anti-inflammatory drug.

Three trials compared the effect of ginger and NSAID on pain severity; the pooled data of two trial [14,15] indicated that ginger and NSAID were equally effective in reducing the pain severity among women with primary dysmenorrhea, and there was no statistical difference between the two (RR = 1.15, 95% CI = 0.53-2.52, P = 0.72, I2 = 77%) (Figure 4).

ii. Pain duration

**Figure 5:**
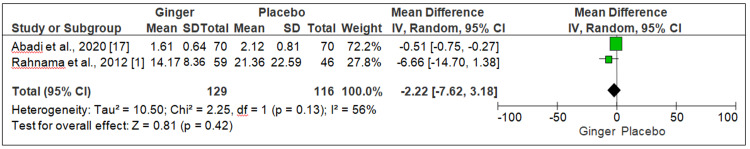
Forest plot ginger versus placebo (pain duration)

Ginger versus placebo: A pooled analysis of Rahnama et al. and Abadi et al. showed the effectiveness of ginger and placebo on pain duration in women with primary dysmenorrhea and revealed no significant difference between ginger and placebo in pain duration in a three days’ regime (MD = -2.22, 95% CI = -7.62-3.18, P = 0.42, I2 = 56%) (Figure 5).

Secondary outcome

We were not able to conduct a meta-analysis of secondary outcomes described in our protocol due to insufficient data. However, we did a small narrative synthesis on the side effects of ginger.

Side effects of ginger

Regarding side effects, the included studies recommend that ginger is generally safe and can cause minor ailments in a very few participants (indigestion and headache). This information is reliable in context to the previous studies on ginger that it has a good safety profile when utilized properly. Rahnama et al. [1], Abadi et al. [17] and Janebi et al. [19] have reported headache and heartburn as the side effects of ginger [1,16,18].

We also wanted to reveal statistical evaluation on the effect of ginger in the specific cycles of menstruation. However, due to much fewer studies and excessive heterogeneity, we could not include it in our meta-analysis.

## Discussion

This meta-analysis summarizes the evidence from eight RCT analyzing the effectiveness of ginger on primary dysmenorrhea. This review provides evidence that ginger can eliminate pain related to primary dysmenorrhea. It is consistent with the several findings from the observed studies; we can state that ginger might be considered to treat menstrual pain. Inclusively, ginger was found to be more powerful for relieving pain than placebo, though we have found ginger and NSAID to be equally effective on pain severity. However, these results must be viewed with great caution because of a limited number of experiments, low methodological consistency, and high heterogeneity in the trial.

Various trials have considered ginger for alleviating pain and inflammation. Several reviews support ginger for its potency in pain relief related to auto-immune disease, rheumatoid arthritis, osteoarthritis, burn injury, migraine headache, and constant lower back pain [21-26]. Intake of ginger in folk medicine for the treatment of cold, fever, sore throat, nausea, stomach upset, muscle aches, and arthritis has been documented in various studies [27]. Ginger is effective for several types of aches. However, it is not entirely effective for all pain.

This review also revealed the outcome of ginger on pain duration, although we have found only two studies that assessed pain duration in their trial [1,17]. Abadi et al.’s study [17] found that the length of pain in the ginger group was substantially shorter relative to the placebo group. The results of another study showed that taking ginger was significantly better at reducing the severity of the pain two days before the onset of the menstrual cycle. Yet, in our meta-analysis, we could not find any significant difference between the ginger and placebo groups in the reduction of pain duration.

Rahnama et al.’s study [1] indicates that ginger is comparatively safe with recorded side effects when considering safety (heartburn and headache). According to the Lakhan et al.’s reports [28], ginger encompasses an excellent safety profile when consumed precisely. A systematic analysis indicates that ginger has a higher safety profile than NSAIDs for pain relief, with a smaller number of gastric side effects and fewer kidney risks.

The conclusion of this review is somewhat the same as the previous two reviews on the effect of ginger on primary dysmenorrhea by Daily et al. and Chen et al. [11,12], but our review is different from these two reviews as we have included only RCTs and one more outcome (pain duration) in our review. Daily et al. [11] has used two non-RCT studies by Halder et al. and Gupta et al. [29,30]. Chen et al. has also extensively utilized Halder et al.’s study in their review [12]. Daily et al.’s systematic review [11] reported more supportive findings regarding the effectiveness of ginger for primary dysmenorrhea than Chen et al.’s systematic review [12]. The included trials had a "low or moderate" risk of bias according to Daily et al.’s assessment. Ginger was "highly useful in the reduction of primary dysmenorrhea," according to their review. In difference, Chen et al.’s review included trials that had “high-risk bias.” Chen et al.’s effect size for ginger against placebo was lower than Daily et al.’s effect size.

This review has shown that ginger can minimize pain in one or two periods. The present analysis provides compelling proof of the impact of ginger on relieving menstrual pain. Based on beneficial effects and minimal side effects, ginger may be a potential adjunct treatment for primary dysmenorrhea. There is a great requirement to enhance the practical or methodological consistency of upcoming studies. Future studies have to use efficient methods for generating random sequences, allocation concealment, blinding participants, blinding outcome assessors, resolving missing data (use of intent-to-treat analysis), and reporting on pre-specified findings.

The standard of this meta-analysis was an effort to incorporate the existing accessible facts sustaining the effectiveness of ginger in the treatment of primary dysmenorrhea. To eliminate errors, data extraction, quality assessment, and study selection were done separately by three authors. However, following are some of the drawbacks that have been found. First, only RCTs have been included in the study, not observational studies, which can limit our study's sample size. Second, all the studies were carried out in Iran, which could have an impact on the generalizability of the findings. Third, the high heterogeneity between the trials and consequences of our review must be analyzed with caution. Effective design, properly powered, and prolonged RCTs are required in order to assess the impact of ginger on primary dysmenorrhea. Fourth, this review was not cataloged on PROSPERO (International Prospective Register of Systematic Reviews); however, upcoming trials with big sample sizes should use the registration system.

## Conclusions

The finding in this study has verified the possibility of ginger efficacy in the treatment of primary dysmenorrhea, though no/small side effects have been identified and its use is associated with health benefits. Ginger is easily accessible due to its low cost. It can also be commonly used in the treatment of primary dysmenorrhea. The use of ginger is very useful and effective as NSAIDs because of the increasing trend in the use of traditional medicine and herbal medicine, particularly for people who do not want to use chemical drugs with more side effects. We strongly recommend that further research be performed with a greater number of patients regarding the effectiveness and protection of various doses of ginger.

## Appendices

PubMed search strategies

Filter Applied: 2000-2020, English Language, Full Text Articles, Randomized Controlled Trials

(("ginger"[MeSH Terms] OR "ginger"[All Fields] OR "gingers"[All Fields] OR "ginger s"[All Fields]) AND ("dysmenorrhea"[MeSH Terms] OR "dysmenorrhea"[All Fields] OR "dysmenorrheas"[All Fields] OR "dysmenorrhoea"[All Fields]) AND (("primaries"[All Fields] OR "primary"[All Fields]) AND ("dysmenorrhea"[MeSH Terms] OR "dysmenorrhea"[All Fields] OR "dysmenorrheas"[All Fields] OR "dysmenorrhoea"[All Fields] [All Fields] OR "secondaries"[All Fields] OR "secondary"[MeSH Subheading] OR "secondary"[All Fields]) AND ("dysmenorrhea"[MeSH Terms] OR "dysmenorrhea"[All Fields] OR "dysmenorrheas"[All Fields] OR "dysmenorrhoea"[All Fields]))

("zingiber"[All Fields] OR ("ginger"[MeSH Terms] OR "ginger"[All Fields] OR "gingers"[All Fields] OR "ginger s"[All Fields]) OR ("ginger"[MeSH Terms] OR "ginger"[All Fields] OR ("zingiber"[All Fields] AND "officinale"[All Fields]) OR "zingiberofficinale"[All Fields])) AND (("primaries"[All Fields] OR "primary"[All Fields]) AND ("dysmenorrhea"[MeSH Terms] OR "dysmenorrhea"[All Fields] OR "dysmenorrheas"[All Fields] OR "dysmenorrhoea"[All Fields]))

("ginger"[MeSH Terms] OR "ginger"[All Fields] OR "gingers"[All Fields] OR "ginger s"[All Fields]) AND (("primaries"[All Fields] OR "primary"[All Fields]) AND ("dysmenorrhea"[MeSH Terms] OR "dysmenorrhea"[All Fields] OR "dysmenorrheas"[All Fields] OR "dysmenorrhoea"[All Fields]))

("dysmenorrhea"[MeSH Terms] OR "dysmenorrhea"[All Fields] OR ("painful"[All Fields] AND "period"[All Fields]) OR "painful period"[All Fields]) AND ("ginger"[MeSH Terms] OR "ginger"[All Fields] OR "gingers"[All Fields] OR "ginger s"[All Fields])

("controlled trial"[All Fields] AND "randomized"[All Fields] AND ("dysmenorrhea"[MeSH Terms] OR "dysmenorrhea"[All Fields] OR ("menstruation"[All Fields] AND "pain"[All Fields]) OR "menstruation pain"[All Fields])) OR ("dysmenorrhea"[MeSH Terms] OR "dysmenorrhea"[All Fields] OR ("period"[All Fields] AND "pain"[All Fields]) OR "period pain"[All Fields])

"controlled trial"[All Fields] AND "randomized"[All Fields] AND ("dysmenorrhea"[MeSH Terms] OR "dysmenorrhea"[All Fields] OR "dysmenorrheas"[All Fields] OR "dysmenorrhoea"[All Fields])

("ginger"[MeSH Terms] OR "ginger"[All Fields] OR "gingers"[All Fields] OR "ginger s"[All Fields]) AND ("ginger"[MeSH Terms] OR "ginger"[All Fields] OR "gingers"[All Fields] OR "ginger s"[All Fields] OR (("primaries"[All Fields] OR "primary"[All Fields]) AND ("dysmenorrhea"[MeSH Terms] OR "dysmenorrhea"[All Fields] OR "dysmenorrheas"[All Fields] OR "dysmenorrhoea"[All Fields])))

"ginger"[All Fields]"zingiberofficinale"[All Fields]"primary dysmenorrhea"[All Fields]

(("menstruation pain"[All Fields] AND "randomized"[All Fields]) AND "placebo"[All Fields]) AND "controlled trial"[All Fields]"menstruation pain"[All Fields]

(("effect"[All Fields] OR "effecting"[All Fields] OR "effective"[All Fields] OR "effectively"[All Fields] OR "effectiveness"[All Fields] OR "effectivenesses"[All Fields] OR "effectives"[All Fields] OR "effectivities"[All Fields] OR "effectivity"[All Fields] OR "effects"[All Fields]) AND ("ginger"[MeSH Terms] OR "ginger"[All Fields] OR "gingers"[All Fields] OR "ginger s"[All Fields]) AND ("officinale"[All Fields] OR "officinales"[All Fields])) AND "primary dysmenorrhea"[Title ("effect"[All Fields] OR "effecting"[All Fields] OR "effective"[All Fields] OR "effectively"[All Fields] OR "effectiveness"[All Fields] OR "effectivenesses"[All Fields] OR "effectives"[All Fields] OR "effectivities"[All Fields] OR "effectivity"[All Fields] OR "effects"[All Fields]) AND "zingiber"[All Fields] AND ("primaries"[All Fields] OR "primary"[All Fields]) AND ("dysmenorrhea"[MeSH Terms] OR "dysmenorrhea"[All Fields] OR "dysmenorrheas"[All Fields] OR "dysmenorrhoea"[All Fields])

("effect"[All Fields] OR "effecting"[All Fields] OR "effective"[All Fields] OR "effectively"[All Fields] OR "effectiveness"[All Fields] OR "effectivenesses"[All Fields] OR "effectives"[All Fields] OR "effectivities"[All Fields] OR "effectivity"[All Fields] OR "effects"[All Fields]) AND ("ginger"[MeSH Terms] OR "ginger"[All Fields] OR "gingers"[All Fields] OR "ginger s"[All Fields])) AND "primary dysmenorrhea"[Title]

"randomized"[All Fields] AND "trial"[All Fields] AND "ginger"[All Fields] AND ("mensuration"[All Fields] AND "pain"[All Fields])

EMBASE search strategies

1. dysmenorrhea AND (pain AND modalities)(randomized trial)) AND (ginger)) AND (mensuration pain) 69

2. (Primary OR dysmenorrhea) AND (ginger OR pharmacological) 652

3. (zingiber AND officinale) AND (dysmenorrhea OR period) AND pain (ginger) AND (dysmenorrhea)) AND (primary dysmenorrhea)) NOT (secondary dysmenorrhea) 704

4. (zingiber AND officinale) AND dysmenorrhea 14

5. zingiber AND officinale (zingiber) OR (ginger)) OR (zingiberofficinale)) AND (primary dysmenorrhea) 2,030

6. ('ginger' OR zingiber) AND (primary AND dysmenorrhea) 34

7. *ginger OR period pain) 34
